# Finite Element Analysis of Stress Distribution in Pediatric Zirconia Crowns Luted With Four Different Cements

**DOI:** 10.7759/cureus.82694

**Published:** 2025-04-21

**Authors:** Subhashree Sahoo, Senthil Kumar Palanimuthu, Ganesh Rajendran, Subbalekshmi T, SelvaKumar Haridoss, Kavitha Swaminathan

**Affiliations:** 1 Pediatric and Preventive Dentistry, Sri Aurobindo College of Dentistry, Sri Aurobindo University, Indore, IND; 2 Pedodontics and Preventive Dentistry, Primary Health Care Corporation, Doha, QAT; 3 Pediatric Dentistry and Preventive Dentistry, Al-Baha University, Al-Baha, SAU; 4 Pediatric Dentistry, Malabar Dental College and Research centre, Edappal, IND; 5 Pedodontics and Preventive Dentistry, Sri Ramachandra Institute of Higher Education and Research, Chennai, IND

**Keywords:** biomechanical analysis, crown retention, finite element analysis, luting cements, occlusal load simulation, pediatric dentistry, primary teeth, stress distribution, zirconia crown

## Abstract

Background

Prefabricated zirconia crowns are increasingly used in pediatric dentistry due to their aesthetic and mechanical advantages. The selection of luting cement significantly influences stress distribution at the crown-tooth interface, potentially affecting long-term clinical performance.

Objective

To evaluate and compare the stress distribution in prefabricated zirconia crowns luted with four different cements zinc phosphate cement (ZPC), zinc polycarboxylate cement (PC), glass ionomer cement (GIC), and resin cement (RC) using finite element analysis (FEA).

Methods

A three-dimensional finite element model of a primary mandibular second molar restored with a prefabricated zirconia crown was developed. The model was analyzed under vertical, oblique, and lateral masticatory loads using four types of luting cements. Stress distribution was assessed using von Mises stress criteria across the tooth, crown, and cement layers.

Results

ZPC exhibited the highest von Mises stress values across all loading conditions, especially in the cement and dentin layers.PC demonstrated the lowest stress values, indicating reduced mechanical strain on the underlying structures.

Conclusion

The choice of luting cement plays a critical role in stress distribution beneath zirconia crowns. Zinc polycarboxylate cement showed the most favorable biomechanical behavior in this finite element model under simulated masticatory forces. However, clinical studies are needed to validate these findings and assess long-term outcomes.

## Introduction

Dental caries remains one of the most prevalent chronic diseases affecting nearly 50% of children worldwide, involving both primary and permanent teeth [1]. Particularly in younger children, caries in the posterior teeth can result in extensive structural loss, necessitating full-coverage restorations.

Traditionally, stainless steel crowns (SSCs) have been the gold standard for restoring extensively decayed primary molars due to their durability and ease of placement. However, esthetic limitations and growing parental demand for natural-looking restorations have led to increased use of prefabricated zirconia crowns (PZCs)​ [2]. These crowns provide superior esthetics, high fracture resistance, and biocompatibility, making them a favorable alternative in pediatric restorative dentistry​ [3]. The longevity and retention of PZCs depend not only on crown adaptation but also on the mechanical interaction with the underlying luting cement. Various cements - including zinc phosphate (ZPC), zinc polycarboxylate (PC), glass ionomer cement (GIC), and resin cements(RC) - are used to lute PZCs, each with unique physical properties influencing stress distribution ​[4]. Improper stress distribution may lead to microfractures in the crown or dislodgement over time, compromising clinical success.

Finite element analysis (FEA) is a well-established computational method that allows simulation of mechanical behavior within complex dental structures, enabling assessment of stress and strain patterns in a highly controlled virtual environment​ [5]. Previous FEA studies have evaluated crown and cement interactions in adult teeth; however, biomechanical data specific to primary molars restored with zirconia crowns remain limited.

Given the increasing popularity of PZCs and the variability in cement selection, it is essential to understand how different luting agents affect stress distribution at the crown-tooth interface in pediatric applications. Unlike prior studies that focused on molars or cement thickness, our study compares four distinct cements using a validated pediatric crown model. This study aims to evaluate and compare the stress distribution patterns in pediatric zirconia crowns luted with four different cements using finite element analysis. The insights gained from this study may assist clinicians in selecting the most biomechanically favorable luting cement for prefabricated zirconia crowns in pediatric patients, thereby potentially enhancing crown longevity and minimizing structural failures.

## Materials and methods

Ethical approval

The study received ethical clearance from the Institutional Ethical Committee (IEC), reference number CSP/22/SEP/116/498.

Finite element model development

**Figure 1:**
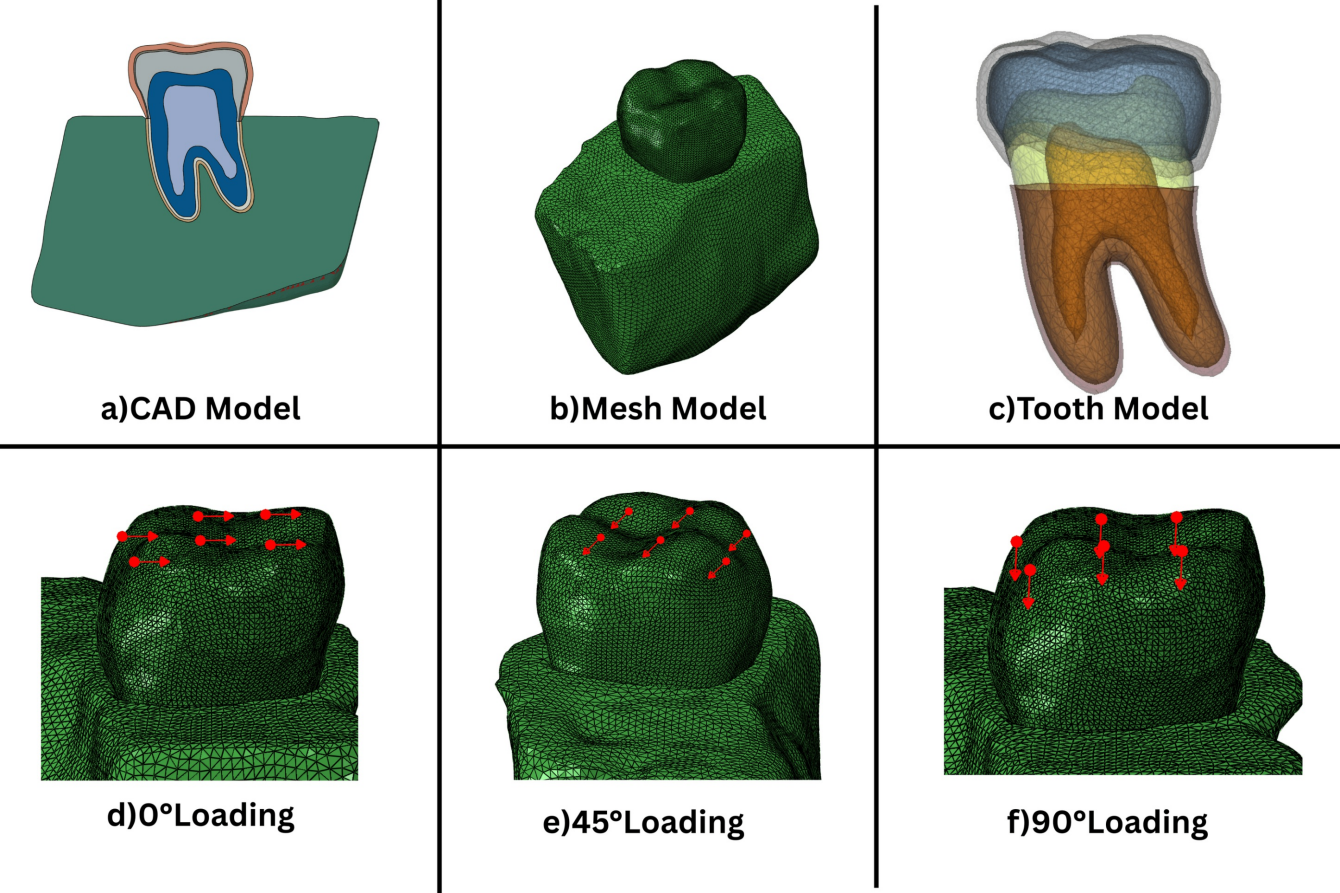
Finite element modeling workflow and simulated loading conditions. (a) Three-dimensional Computer Aided Design (CAD) model of the primary mandibular second molar with zirconia crown adaptation; (b) Meshed finite element model showing boundary definitions; (c) Sectional view of the layered tooth anatomy including enamel, dentin, and pulp; (d) Vertical occlusal loading applied at 0°; (e) Oblique loading applied at 45°; (f) Lateral loading applied at 90°. Red arrows indicate the direction and location of masticatory force application.

A three-dimensional model of a primary mandibular second molar was reconstructed using high-resolution cone beam computed tomography (CBCT) data (Planmeca ProMax 3D, Planmeca, Helsinki, Finland), previously validated in a pediatric molar finite element analysis study (Kirthiga et al., 2018) [6]. The scan was converted to a stereolithographic (STL) file and segmented using MIMICS 19.0 (Materialise, Leuven, Belgium), followed by anatomical reconstruction in SolidWorks 2021 (Dassault Systèmes, Cedex, France), isolating enamel, dentin, pulp, periodontal ligament (PDL), cortical bone, and cancellous bone layers. Tooth dimensions were consistent with average morphometric values of mandibular second primary molars reported in pediatric anatomical datasets. To simulate a full-coverage restoration, standardized tooth reduction of 1.5 mm occlusally and 0.8 mm axially was performed virtually, followed by adaptation of a prefabricated zirconia crown (NuSmile ZR, NuSmile Ltd., Houston, TX, USA) modeled with a uniform wall thickness of 0.78 mm using Abaqus 6.14 (Dassault Systèmes, Cedex, France). A uniform cement layer of 50 µm thickness was applied between the tooth and crown interfaces in all models [7-9]. The final Computer Aided Design (CAD) assembly included detailed contact interfaces and layer-specific structures for each material (Figure 1).

Material properties

Four distinct luting agents were analyzed: zinc phosphate cement (ZPC), zinc polycarboxylate cement (PC), glass ionomer cement (GIC), and resin cement (RC). These materials were selected due to their widespread clinical use in pediatric dentistry and their distinct mechanical profiles that influence stress transmission patterns. Material properties, including Young’s modulus and Poisson’s ratio for each model component, were derived from validated peer-reviewed studies [10] and are summarized in Table 1. No mechanical testing was performed; values were literature-sourced for consistency and reproducibility.

**Table 1:** Material properties assigned to finite element model components Source: Prabhakar et al. [10]

Component	Young's Modulus (MPa)	Poisson's Ratio
Enamel	8,400	0.31
Dentine	18,600	0.31
Cementum	18,600	0.31
Cortical	13,700	0.3
Cancellous	1,370	0.3
Pulp	0.010	0.49
Periodontal Ligament	0.667	0.49
Zirconia Crown	242,000	0.26
Zinc Phosphate	22,400	0.35
Glass Ionomer Cement	12,000	0.25
Zinc Polycarboxylate	5,110	0.35
Resin Cement	7000	0.27

Meshing and convergence testing

Meshing was conducted in HyperMesh 14 (Altair Engineering, Troy, MI, USA), using second-order 10-node tetrahedral elements. Mesh convergence was validated by refining the mesh until changes in von Mises stress values were <5% between successive refinements. The final mesh included approximately 184,000 to 280,000 elements, depending on the cement type. Node and element counts for each structure are shown in Table 2.

**Table 2:** Mesh details of finite element model

Model Component	No. of Nodes	No. of Elements
Cortical	8455	16844
Cancellous	29260	144537
Cementum	9375	28049
Zirconia Crown	12886	46580
Enamel	14388	63725
Dentine	24396	114453
Periodontal Ligament	9377	28101
Pulp	7843	36482
Cement	9321	27766

Boundary conditions and load application

The outer surfaces of the cortical bone were fixed in all directions to simulate anatomical support and eliminate rigid body motion. Vertical (0°), oblique (45°), and lateral (90°) masticatory loads were applied at five functional contact points on the occlusal surface, mimicking clinically observed load vectors. Each model was tested under both mean occlusal load (311 N) and maximum load (396 N) conditions [11]. Load vectors were distributed equally across contact points.

Stress analysis and evaluation criteria

All simulations were run in Abaqus 6.14 under a linear static analysis protocol. Stress distribution was assessed using von Mises stress criteria, which indicate the risk of structural failure due to equivalent stresses under complex loading conditions. Color-coded contour maps were generated to visualize stress magnitudes within the crown, cement, dentin, and pulp structures. No statistical comparison or inferential testing was performed, as this is a deterministic simulation model.

## Results

In this finite element analysis (FEA) study, von Mises stress values were evaluated for four different luting cements under three loading angles (0°, 45°, and 90°), representing vertical, oblique, and lateral masticatory forces, respectively. A NuSmile ZR zirconia crown was modeled over a primary mandibular second molar, with a uniform cement layer of 50 µm thickness. Stress values were measured across key anatomical structures, and visualized using color-coded maps where red represented maximum stress and blue indicated minimal stress. Mesh convergence testing confirmed solution stability, with less than 5% variation between successive mesh refinements, validating the robustness of the results. As this study used computational finite element analysis, no sample size (N) or percentage (%) values were generated.

**Figure 2:**
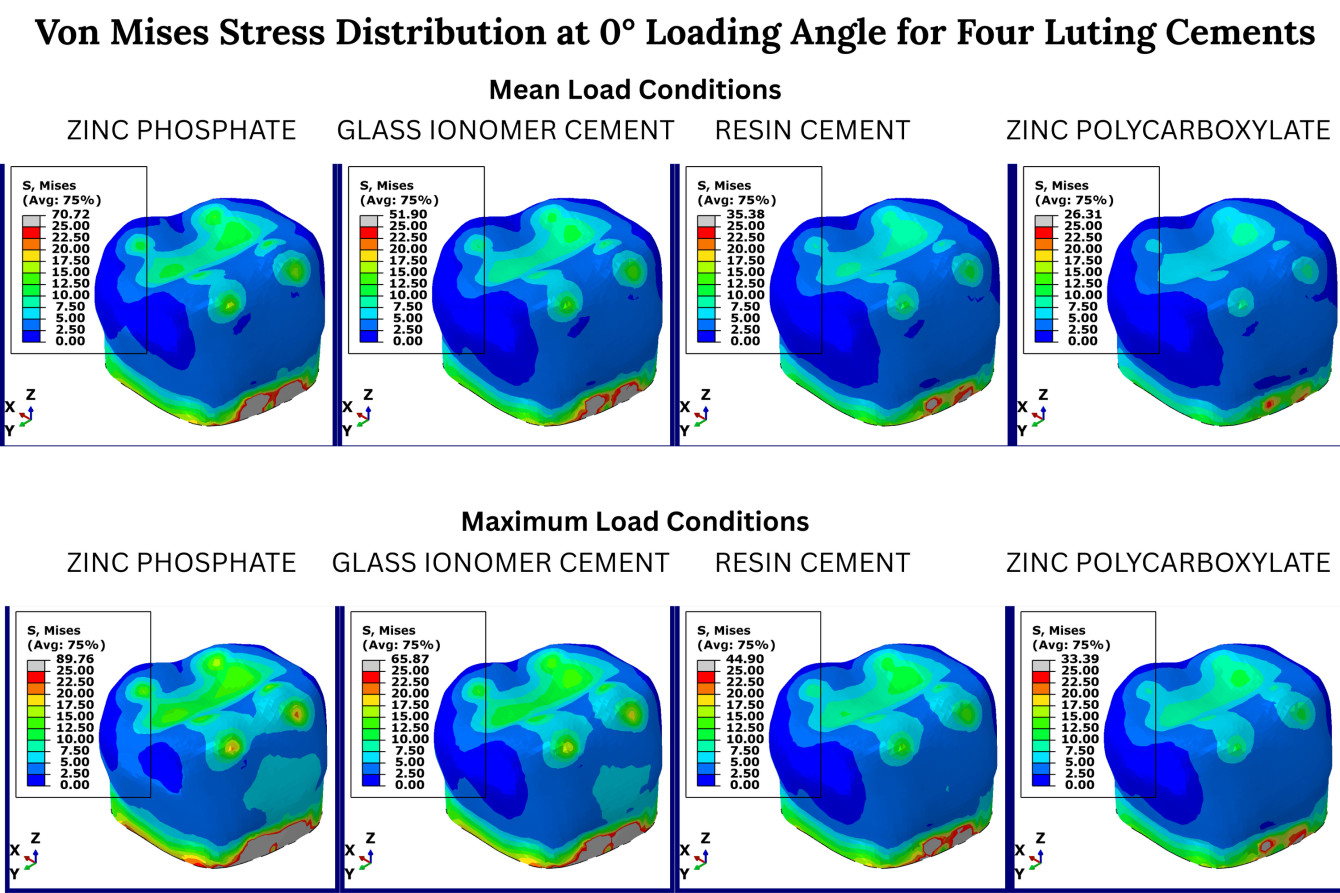
Von Mises stress distribution at 0°loading angle for four luting cements Color-coded von Mises stress maps of prefabricated zirconia crowns cemented with four luting agents, zinc phosphate, glass ionomer, resin, and zinc polycarboxylate under simulated (0°) masticatory loading conditions. The first column of each cement group represents the mean occlusal load, and the second column represents the maximum occlusal load. Red areas indicate maximum stress concentration; blue areas represent minimal stress. Stress values are derived from finite element analysis and are presented in megapascals (MPa). No statistical testing was performed, as this is a deterministic simulation study.

As illustrated in Figure 2, under 0° vertical loading, stress was predominantly concentrated in the cervical region of the crown and cement layer, particularly in the ZPC group. Mean loading generated lower stress intensity across all materials, with the PC group showing the most uniform stress distribution.

**Figure 3:**
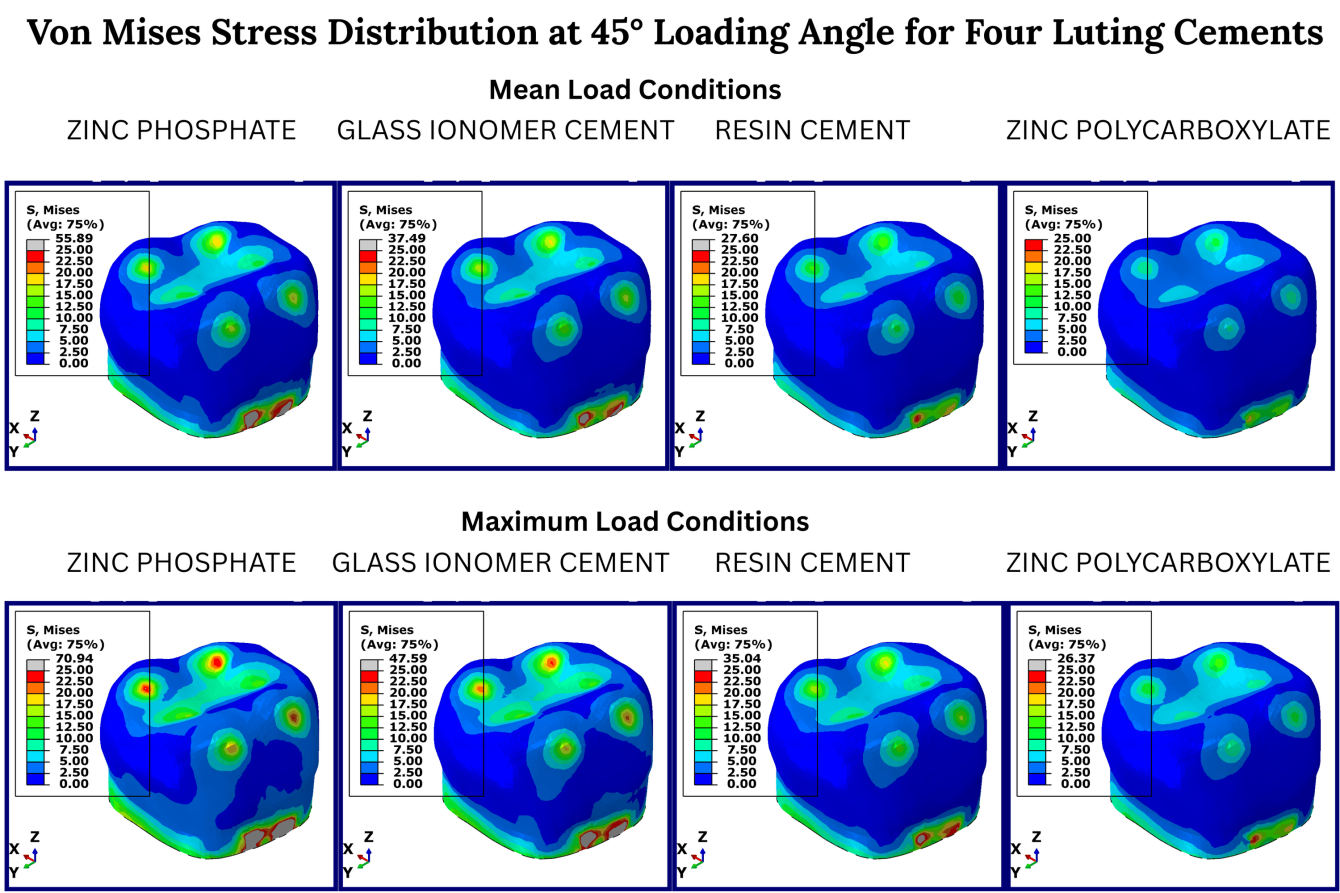
Von Mises stress distribution at 45° loading angle for four luting cements Color-coded von Mises stress maps of prefabricated zirconia crowns cemented with four luting agents, zinc phosphate, glass ionomer, resin, and zinc polycarboxylate under simulated (45°) masticatory loading conditions. The first column of each cement group represents the mean occlusal load, and the second column represents the maximum occlusal load. Red areas indicate maximum stress concentration; blue areas represent minimal stress. Stress values are derived from finite element analysis and are presented in megapascals (MPa). No statistical testing was performed, as this is a deterministic simulation study.

Under 45° oblique loading (Figure 3), the stress distribution became more asymmetrical, with higher intensity in the mesiobuccal and distolingual regions. This loading angle magnified differences between cements, with the ZPC group again demonstrating the highest stress peaks, while polycarboxylate cement maintained lower transmission to dentin and pulp layers.

**Figure 4:**
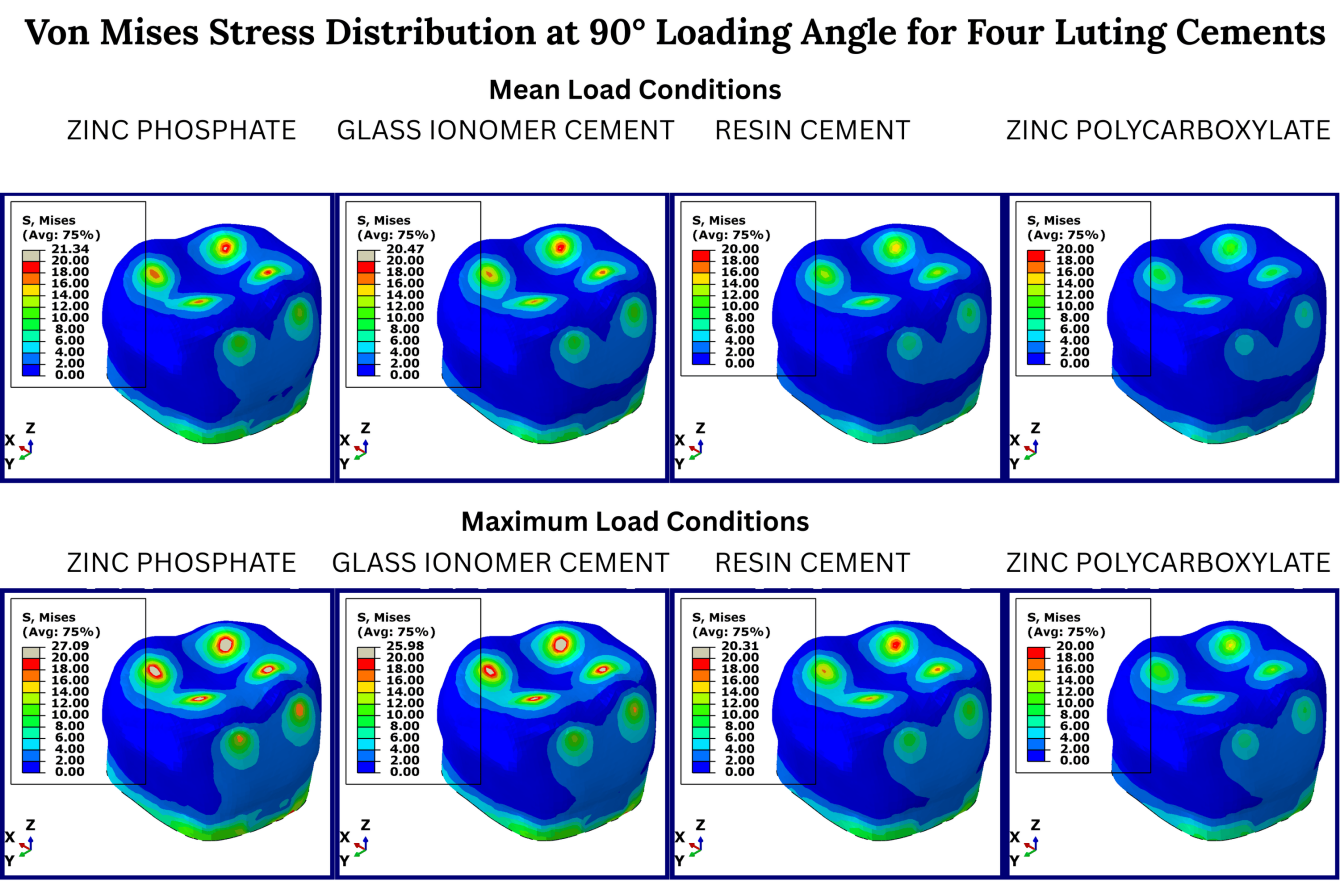
Von Mises stress distribution at 90° loading angle for four luting cements Color-coded von Mises stress maps of prefabricated zirconia crowns cemented with four luting agents, zinc phosphate, glass ionomer, resin, and zinc polycarboxylate under simulated (90°) masticatory loading conditions. The first column of each cement group represents the mean occlusal load, and the second column represents the maximum occlusal load. Red areas indicate maximum stress concentration; blue areas represent minimal stress. Stress values are derived from finite element analysis and are presented in megapascals (MPa). No statistical testing was performed, as this is a deterministic simulation study.

Lateral loading at 90° (Figure 4) resulted in the highest crown deflection patterns. The inner crown and cement interface exhibited distinct peak stresses, especially under maximum load. RC and GIC produced intermediate stress patterns, while PC continued to show favorable dissipation of stress throughout the supporting tooth structures.

Stress distribution under mean load conditions

Table 3 summarizes the stress values recorded under mean masticatory loads.

**Table 3:** Stress result summary under mean load conditions (MPa) Stress values are represented in megapascals (MPa) derived from a single finite element simulation per condition. As finite element analysis (FEA) is a deterministic model-based analysis, statistical significance testing was not performed. P-values and test statistics (e.g., t, F, chi-square) were not applicable as this study did not involve hypothesis testing or repeated sampling. All stress values were derived from static FEA simulations under controlled loading conditions.

Name	Zinc Phosphate			Glass Ionomer Cement			Resin Cement			Zinc Polycarboxylate Cement		
Loading Degree	0	45	90	0	45	90	0	45	90	0	45	90
Cortical	33.55	25.27	18.23	33.55	25.27	18.23	33.56	25.27	18.23	33.56	25.27	18.23
Cancellous	20.86	14.47	11.1	20.86	14.47	11.1	20.86	14.47	11.1	20.86	14.47	11.1
Cementum	60.27	39.16	21.69	59.64	38.92	21.54	59.27	38.65	21.36	59.18	38.45	21.23
Zirconia Crown	517.2	513.77	574.83	520.4	517.53	577.38	522.81	520.54	579.14	523.43	521.08	579.49
Inner Crown	979.77	607.66	137.82	992.59	616.59	138.22	1003.15	624.02	138.53	1008.45	627.8	138.59
Dentine	353.12	228.18	41.28	347.93	224.83	40.57	344.25	222.49	40.06	342.83	221.62	39.85
Periodontal Ligament	3.28	2.05	0.54	3.28	2.05	0.54	3.28	2.05	0.54	3.28	2.05	0.54
Pulp	2.33E-05	1.39E-05	1.38E-05	2.32E-05	1.39E-05	1.38E-05	2.31E-05	1.39E-05	1.38E-05	2.30E-05	1.40E-05	1.38E-05
Cement	70.72	55.89	21.34	51.90	37.49	20.47	35.38	27.6	16.01	26.31	20.77	12.29

The highest stress concentrations were consistently observed in the ZPC group, particularly in the cement layer (70.72 MPa at 0°) and inner crown (979.77 MPa at 0°). In contrast, the lowest stress values were associated with PC, especially in the cement (12.29 MPa at 90°) and dentin (39.85 MPa at 90°) layers.

The stress within the zirconia crown increased progressively across loading angles, while RC and PC groups showed slightly higher crown stress but lower cemental stress compared to ZPC. Notably, stress values in the pulp remained negligible across all cements, with readings below 2.40E-05 MPa, indicating minimal mechanical impact on pulpal tissues. The cement layer stress in the ZPC group was more than 2.6 times higher than that observed with PC under vertical load (70.72 MPa vs. 26.31 MPa at 0°) (Figure 2).

Stress distribution under maximum load conditions

Under peak occlusal forces (Table 4), the same trends persisted, with ZPC consistently producing the highest stress values in both the cement and inner crown.

**Table 4:** Stress result summary under maximum load conditions (MPa) Stress values are represented in megapascals (MPa) derived from a single finite element simulation per condition. As finite element analysis (FEA) is a deterministic model-based analysis, statistical significance testing was not performed. P-values and test statistics (e.g., t, F, chi-square) were not applicable, as this study did not involve hypothesis testing or repeated sampling. All stress values were derived from static FEA simulations under controlled loading conditions.

Name	Zinc Phosphate			Glass Ionomer Cement			Resin Cement			Zinc Polycarboxylate Cement		
Loading Degree	0	45	90	0	45	90	0	45	90	0	45	90
Cortical	42.58	32.08	23.13	42.59	32.08	23.13	42.59	32.08	23.13	42.59	32.08	23.13
Cancellous	26.47	18.36	14.09	26.47	18.36	14.09	26.47	18.36	14.09	26.47	18.36	14.09
Cementum	76.49	49.7	27.54	75.7	49.4	27.34	75.23	49.06	27.11	75.11	48.81	26.94
Zirconia Crown	656.44	652.09	729.59	660.51	565.87	732.83	663.56	660.69	735.06	664.36	661.37	735.51
Inner Crown	1243.56	771.26	174.93	1259.82	782.6	175.43	1273.22	792.03	175.83	1279.95	796.83	175.91
Dentine	448.2	289.62	52.39	441.6	285.36	51.5	436.94	282.39	50.84	435.13	281.29	50.57
Periodontal Ligament	4.16	2.6	0.69	4.16	2.6	0.69	4.16	2.6	0.69	4.16	2.6	0.69
Pulp	2.96E-05	1.76E-05	1.76E-05	2.95E-05	1.77E-05	1.75E-05	2.93E-05	1.77E-05	1.75E-05	2.92E-05	1.77E-05	1.75E-05
Cement	89.76	70.94	27.09	65.87	47.59	25.98	44.90	35.04	20.31	33.39	26.37	15.6

The cement stress reached 89.76 MPa in ZPC compared to just 15.6 MPa in the PC group under lateral load (90°). The inner crown stress peaked at 1279.95 MPa in the PC group at 0°, possibly indicating increased load transmission beyond the cement interface.

Dentin and periodontal ligament (PDL) stress levels were higher under maximum load but followed consistent comparative trends across all cement types. The pulp layer remained unaffected, with stress levels remaining in the range of 2.92E-05-2.96E-05 MPa.

These findings emphasize that PC consistently resulted in the lowest stress transmission to surrounding structures, potentially offering biomechanical advantages in clinical applications.

## Discussion

This finite element analysis (FEA) study aimed to evaluate the biomechanical behavior of prefabricated zirconia crowns (PZCs) in primary mandibular molars when cemented using four different types of luting agents under varying masticatory loads. This study focused exclusively on the primary mandibular second molar, as it plays a pivotal role in mastication, space maintenance, and arch integrity during the mixed dentition phase. Moreover, due to its anatomical similarity to permanent molars and frequent indication for full-coverage restorations, it provides a relevant and clinically significant model for evaluating stress behavior in pediatric zirconia crowns. The results clearly indicated that zinc polycarboxylate cement (PC) consistently transmitted the lowest von Mises stress across all evaluated dental structures, whereas zinc phosphate cement (ZPC) demonstrated the highest stress concentration, particularly within the cement layer and inner crown interface.

Our findings align with the conclusions reported by Waly et al. (2021), who observed that stiffer cements like ZPC elevated stress levels within the tooth structure while reducing deformation in the crown itself​ [12]. This trade-off is critical in pediatric dentistry, where preserving dentin and minimizing pulpal trauma is a priority. The observed minimal stress in pulp tissues across all cement types in our study reinforces previous claims that PZCs, when properly adapted, are biomechanically safe for pediatric use.

Interestingly, the present study further supports the findings of Chung et al. (2022), who demonstrated through FEA that cement type had a greater influence than cement thickness on overall stress distribution in PZCs​ [4]. Our results also confirm their assertion that resin-based cements, while offering high retention, may exhibit higher stress values than polycarboxylate alternatives. In our model, resin cements showed intermediate stress levels, suggesting a balance between strength and flexibility.

The performance of zinc polycarboxylate cement (PC) in minimizing stress transfer could be attributed to its modulus of elasticity, which more closely resembles that of natural dentin. This allows for more favorable stress dispersion under occlusal loads. This observation is supported by the systematic review conducted by Alzanbaqi et al. (2022), which noted the biocompatibility and marginal integrity benefits of tooth-colored restorations like PZCs when paired with appropriate cements​ [13].

From a clinical perspective, these findings reinforce the recommendation to individualize cement selection based on the biomechanical needs of the restored tooth. While resin cements are widely advocated for their bonding capabilities and retention strength, zinc polycarboxylate may offer a biomechanical advantage, particularly in young children with minimal remaining dentin in high-stress areas.

Moreover, Iampinitkul et al. (2024) highlighted that microleakage and long-term cement degradation remain critical concerns for PZCs, especially due to their prefabricated, non-customizable design​ [14]. Our study, while not directly evaluating microleakage, indirectly supports this concern by showing that higher stress values in the cement layer (e.g., ZPC group) may correlate with higher risks of marginal failure.

Strength of this study

This study presents several noteworthy strengths. First, it utilizes a validated three-dimensional finite element model of the primary mandibular second molar, which closely mimics the anatomical complexity and clinical conditions encountered in pediatric restorative dentistry. Second, by evaluating four commonly used luting cements, zinc phosphate, zinc polycarboxylate, resin, and glass ionomer, the study offers a comprehensive comparison of stress behavior under clinically relevant masticatory loads. The simulation includes vertical, oblique, and lateral forces, reflecting real-world functional conditions in mixed dentition. Additionally, the methodology incorporates precise material properties, standardized cement thickness, and rigorous mesh validation to enhance the accuracy and reproducibility of the results. By focusing on von Mises stress distribution across various tooth structures, the findings provide meaningful biomechanical insights that can aid clinicians in selecting luting agents for zirconia crown placement in pediatric patients.

Limitations

However, the study also has notable limitations. The simulation assumes that all materials are homogeneous, isotropic, and linearly elastic, which does not fully represent the complexity and variability of biological tissues. Real-world variations in tooth anatomy, cement layer thickness, and dynamic occlusal forces are not incorporated, limiting direct clinical generalizability. Furthermore, the FEA results have not been validated through experimental or clinical studies, which may reduce the external validity of the findings. Lastly, while masticatory loading conditions were modeled in three directions, the boundary conditions may not fully mimic the range of forces encountered in pediatric mastication, warranting cautious interpretation of the results.

Future directions

Further in vitro and clinical studies should explore the long-term performance of these cements, including microleakage analysis over time, retention under cyclic loading, and thermocycling. PZC failure modes across different cement types. Additionally, incorporating CAD/Computer Aided Manufacturing (CAM)-based custom zirconia crowns, as suggested in recent literature, may help address adaptation variability and further optimize stress distribution​.

## Conclusions

Within the limitations of this finite element study, zinc polycarboxylate cement exhibited the most favorable stress distribution pattern, transmitting lower stresses to the tooth structure compared to other luting agents. In contrast, zinc phosphate cement consistently demonstrated higher von Mises stress concentrations, particularly within the cement and inner crown layers. These findings suggest that the choice of luting cement may influence the biomechanical behavior of prefabricated zirconia crowns in pediatric applications. However, as this study is based on computational modeling using idealized, isotropic material properties and static loading conditions, the results should be interpreted as predictive simulations rather than definitive clinical outcomes. Further in vitro or clinical validation is warranted to substantiate these findings and guide evidence-based material selection in pediatric restorative dentistry.
